# Dielectric function and thermo-optic coefficients of silicon-doped GaN substrates at elevated temperature from 298 K to 873 K in the UV-Vis-NIR spectrum

**DOI:** 10.1038/s41598-025-87243-w

**Published:** 2025-02-03

**Authors:** Subiao Bian, Xi Chen, Changcai Cui

**Affiliations:** 1 College of Metrology Measurement and Instrument, China Jiliang University, Hangzhou, 310018 China; 2 Institute of Manufacturing Engineering, Huaqiao University, Xiamen, 361021 China; 3 Dep. Física Aplicada, Feman Group, Universitat de Barcelona, Barcelona, 08028 Spain

**Keywords:** Applied optics, Condensed-matter physics, Techniques and instrumentation, Characterization and analytical techniques, Semiconductors, Surfaces, interfaces and thin films

## Abstract

Understanding the thermal influence on gallium nitride (GaN) single crystal substrates is critical for the advancement of GaN-based optoelectronic devices. In this study, we comprehensively characterized the thermal effects on the optical properties of silicon-doped GaN substrates using spectroscopic ellipsometry over a broad wavelength range from 250 nm to 1600 nm. The dielectric function of GaN was determined at temperatures ranging from 298 K to 873 K, demonstrating consistent temperature-dependent behavior. The exciton transitions were precisely characterized and modeled using the empirical Varshni expression. Moreover, we report, for the first time, the thermo-optic coefficients across the wide spectrum, parameterized using a Sellmeier model. This work significantly expand the GaN optical properties database beyond thin films and provide essential insights for the design and optimization of next-generation GaN-based optoelectronic devices.

## Introduction

Gallium nitride (GaN) possesses exceptional properties such as a wide bandgap, high electron drift velocity, low dielectric constant, and excellent thermal conductivity. These attributes make it highly suitable for applications in power electronics, microwave communication, photovoltaic inverters, and lighting^1–3^. Historically, GaN-based devices primarily relied on GaN thin films grown heteroepitaxially on sapphire substrates. However, the substantial crystallographic and thermal mismatches between the film and substrate significantly increased defect density, thereby degrading device performance^4–6^. In recent years, advancements in the growth and processing technology of bulk single crystal GaN have progressed rapidly^7^. The widely used GaN crystal features a non-centrosymmetric hexagonal (wurtzite) structure, which promotes stable properties and facilitates easy growth. By introducing impurities into GaN (doping), its electrical and optical properties can be optimized for various applications. Among the different doping methods, Silicon (Si) doping results in N-type GaN, significantly enhancing its electrical properties, making it highly suitable for high-power and high-frequency optoelectronic devices, is the most widely utilized^8^.

Applicable operating temperatures for GaN materials range from room temperature to 600 $^{\circ }$C and can be even higher in specific applications, albeit with the risk of oxidation^9,10^. In GaN-based optoelectronic devices, such as laser diodes and optical filters, the junction temperature of a GaN-based laser diode can exceed 100 $^{\circ }$C, the refractive index varies with temperature, a phenomenon known as the thermo-optic effect, which influences the optimal waveguide design of laser diodes and the spectral response of photodetectors^11,12^. To optimize the design of optoelectronic devices, it is essential to understand the optical properties of GaN materials at their application temperatures.

Extensive research on the optical properties of GaN thin films has revealed that, at room temperature, the exciton transition of GaN is approximately 3.4 eV, and its refractive index ranges from 2.3 to 2.9 across the visible spectrum. Considering temperature, the optical properties of GaN can significantly change in terms of exciton transition and refractive index due to factors like thermal expansion of the lattice^5,13–17^. In above reports, however, GaN films deposited on substrates potentially introduce a complex interplay of factors beyond mere temperature effects on the crystal lattice. In this thermally charged environment, GaN films are not only subjected to intrinsic thermal influences on their crystalline structure but also contend with film stress, surface roughness, impurity diffusion, and other related phenomena. These factors collectively impact the optical behavior of GaN films under thermal processing, resulting in interactions that transcend GaN’s inherent optical properties^17,18^. Therefore, it becomes imperative to isolate and mitigate the impact of extrinsic uncertainties associated with GaN film characteristics by investigating GaN single crystal substrates for their intrinsic optical properties. At present, the optical properties of temperature dependence of GaN substrate across a wide spectral and application temperature range is rarely reported, and its thermo-optic response over a broad bandwidth UV-Vis-NIR is not fully explored. Specifically, the complete dielectric function and the derived thermo-optic coefficients, which are crucial for precise optoelectronic device simulations, have yet to be comprehensively documented.

Spectroscopic ellipsometry is a powerful technique for characterizing the optical properties of semiconductors by measuring changes in the polarization state of light after it is reflected from or transmitted through a sample. For samples with the simple geometric structures, such as flat bare substrates, the basic ellipsometric parameters $\it \Psi$ and $\it \Delta$, when used with an appropriate physical model, can accurately determine the dielectric functions of the substrate materials^19,20^. The parameters $\it \Psi$ and $\it \Delta$ are related to the complex ratio of the reflection coefficients for light polarized parallel ($p$) and perpendicular ($s$) to the plane of incidence.

1 $$\begin{aligned} \rho =\frac{R_p}{R_s}=\tan (\psi ) e^{i \Delta }. \end{aligned}$$

In this work, the optical properties of N-type GaN substrates are systematically investigated by the spectroscopic ellipsometry over the wavelength range from 250 nm to 1600 nm at elevated temperatures from room temperature to 873K. The dielectric function of the GaN substrates is firstly determined and the exciton transitions are further extracted through critical points analysis. Additionally, the first-order thermo-optic coefficient is provided for the first time over a wide spectral range. These findings on GaN substrates are analyzed and compared with previous studies on thin films.

## Experiment

Commercially available single-crystal N-type Si-doped GaN substrates (doping concentration of $10^{18} \, \textrm{cm}^{-3}$ and $R < 0.05 \, \Omega \text {cm}$), grown by Hydride Vapor Phase Epitaxy (HVPE) and supplied by Hefei Jinko Material Technology Co., Ltd, were investigated. The GaN substrates possess a hexagonal wurtzite structure ($\alpha$-GaN, uniaxial crystal) with a crystal orientation of $\langle 0001 \rangle \pm 30'$, indicating that the optical axis is perpendicular to the substrate surface. The Ga-face of the substrate was processed by chemical mechanical polishing, achieving a surface roughness of $\le$ 0.5 nm, making it suitable for epitaxial growth.

Ellipsometric spectra of the GaN were obtained using a commercial Mueller matrix ellipsometer (ME-L, Wuhan Yiguang Technology Co., Ltd.). The spectral range cover 250 nm to 1600 nm, with incident angles of $55^{\circ }$, $65^{\circ }$, and $75^{\circ }$. To investigate the high-temperature response of the optical properties of GaN substrates, a multifunctional heating and cooling stage (THMS600, Linkam, UK) was used to control the experimental temperature. The stage covers a temperature range from -196 $^{\circ }$C to 600 $^{\circ }$C, with a heating rate adjustable from 0.1 $^{\circ }$C to 150 $^{\circ }$C per minute, and a temperature control accuracy of 0.1 $^{\circ }$C. The GaN substrate was placed directly on the stage without any protective measures, as the temperatures used in the experiment were below the rapid oxidation threshold for GaN, which typically occurs around 800 $^{\circ }$C^21^.

According to previous reports^22^, GaN exhibits distinct dielectric values (approximately 2 near the bandgap) along the directions parallel $\varepsilon _o$ and perpendicular $\varepsilon _e$ to the optical axis. In principle, the contribution that dominates SE data is the projection of the dielectric tensor along the line formed by the surface and the plane of incidence. When the optical axis is perpendicular to the incident plane, the anisotropy cannot be simply neglected to analyze with the isotropic model, as doing so would result in an overestimation of the bandgap of GaN^15^. However, with the optical axis of GaN being perpendicular to the surface, cross-polarization effects diminish, and the dielectric function along the $p$ and $s$ directions is approximately equal to $\varepsilon _o$^23^. Consequently, an isotropic model can be directly utilized for analysis of ellipsometric data from single surface reflection.

**Fig. 1:**
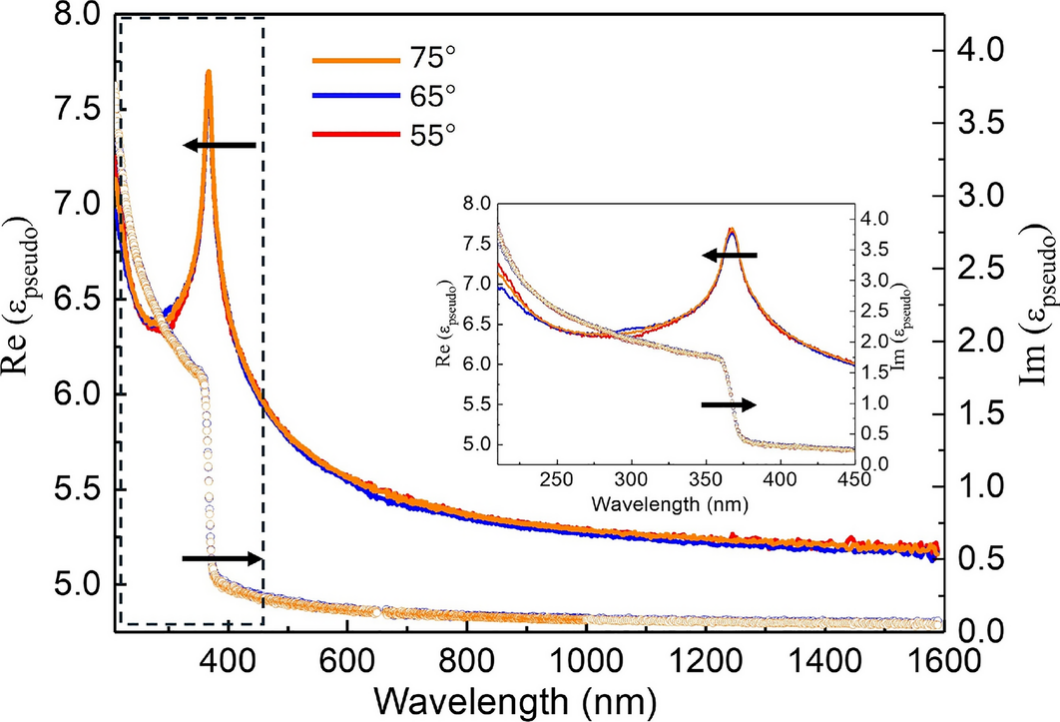
Pseudo dielectric function $\varepsilon _{pseudo}$ of Si doped GaN substrate measured by the spectroscopic ellipsometer at 298 K. The left arrow and right arrow point the real part and imaginary part of the pseudo dielectric function, respectively. The inset shows the zoomed-in view at the wavelength range from 250 nm to 450 nm.

To confirm and exclude the impact of potential anisotropy, the pseudo-dielectric function of the GaN substrate at room temperature (298 K) is presented in Fig. 1. For clarity, the UV portion marked by a black frame is further enlarged and embedded in the figure. Pseudo-dielectric functions at different angles of incidence are often used to assess the presence of an oxide layer and optical anisotropy, as they calculate “dielectric function” values assuming the sample is a bare isotropic substrate without an overlayer and with a perfectly smooth surface^23^. As shown, the pseudo-dielectric functions at three different incident angles almost overlap across the wavelength range, indicating negligible anisotropy. This observation is further validated by the experimental data from our custom-built Mueller matrix ellipsometer^24^. Below the bandgap, the imaginary part of the pseudo-dielectric function exhibits weak absorption, which is not intrinsic to the GaN substrate. This phenomenon is attributed to the chemical mechanical polishing (CMP) process of the semiconductor substrate, which inevitably introduces an ultra-thin amorphous layer, several nanometers thick, on the substrate surface^25^. Consequently, in the subsequent analysis of the ellipsometric parameters, it is essential to consider the presence of this extra ultra-thin overlayer on the GaN substrate.

In analysis of ellipsometric data over the wavelengths, the dielectric function is often parametered by several oscillators to minimize the numbers of fitting parameters. Herein, the dielectric function of the GaN substrate was parametered using a combination of a PSemi-tri oscillator and a Gaussian oscillator, with Kramers-Kronig integration applied. The PSemi-tri oscillator, characterized by seven free parameters, was developed to accurately fit the critical points of direct-bandgap semiconductors, with its center energy located near the absorption edge^26^. The Gaussian oscillator, with three parameters, describes the dielectric behavior of GaN in the deep ultraviolet region. The dielectric function of ultra-thin amorphous layer is parameterized by a Cauchy model. Accurately determining the thickness and refractive index of the ultra-thin film is challenging in ellipsometry measurement. However, our primary goal is to remove the influence of the ultra-thin film on the optical properties of the substrate. Therefore, we fixed the refractive index after fitting it at room temperature. During the heating process, only the thickness of the amorphous layer is considered to change.

The model proposed here is capable of accurately reproducing all ellipsometric parameters of the GaN substrate at elevated temperatures, with a mean square error (MSE) consistently below 2. The refractive index *n* of the amorphous layer was determined using the Cauchy model ($n=A+\frac{B}{\lambda ^{2}}$), with parameters $A = 1.190$ and $B = 0.00722$. The thickness of this layer was observed to decrease from 4.10 nm to 1.1 nm as the temperature increased. It should be noted that this change in the amorphous layer thickness does not represent a true physical evolution but rather a combined effect of changes in both thickness and refractive index. The dielectric function of GaN will be detailed discussed in the following section.

## Discussion

**Fig. 2:**
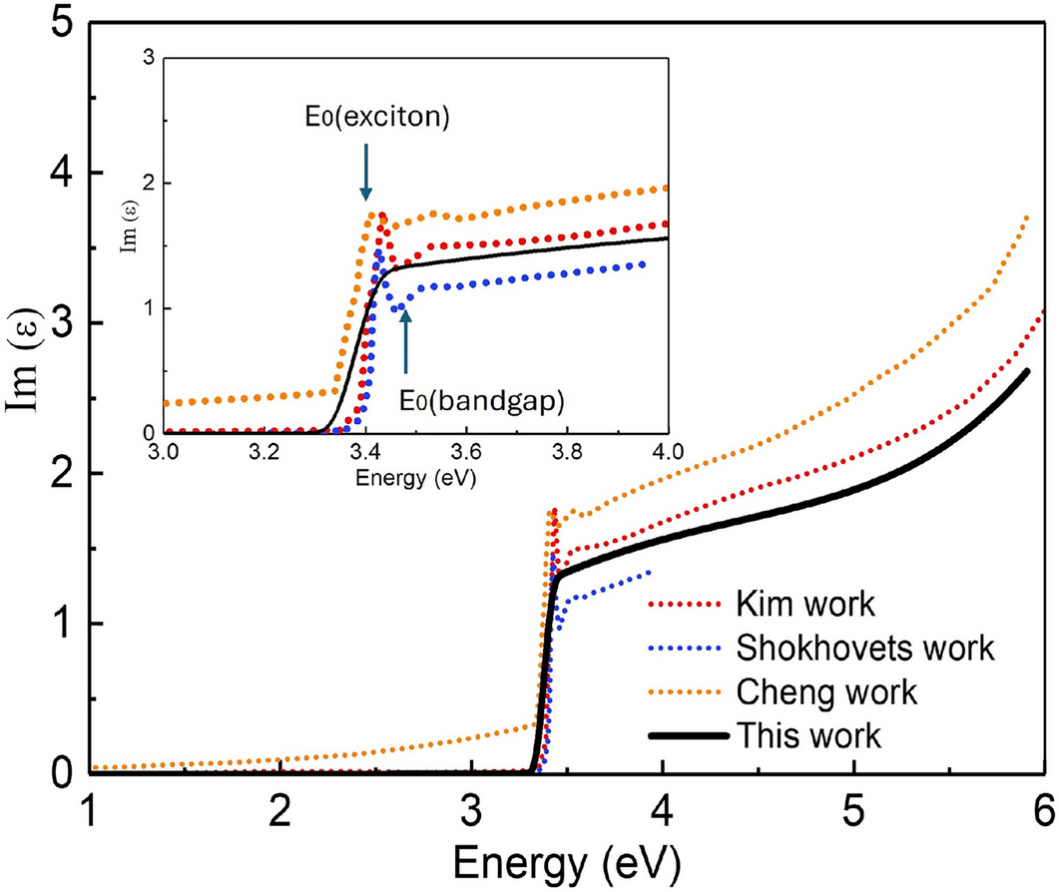
Dielectric function $\varepsilon$ of Silicon doped GaN at 298 K (black line), compared with those of Kim work^13^, Shokhovets work^27^, Cheng work^17^. The inset shows the zoomed-in view in the spectral range of 3-4 eV and the exciton and inter transition is marked by arrow, respectively.

Fig.2 presents the imaginary part of the dielectric spectrum for the Si-doped GaN substrate at 298 K. The measured dielectric functions (black line) show good agreement with the data reported in the literature for undoped GaN thin films^13,17,27^. Interestingly, previous data from undopped GaN thin films can observe two clearly peak near the absorption edge (around 3.4 eV) that had already been attributed to exciton transition and inter-band transition, but hardly-distinct in this work. This is effectively explained that the free electron concentration introduced by doping increases, shielding the coulomb interaction between electrons and holes, thereby inhibiting the formation of exciton as well as the formation of deep level defects that trap carriers, thereby reducing the population of free exciton leading to the disappearance of exciton-related phenomenon.^28–30^. By observation of the relatively small slope of absorption edge of Si-doped GaN substrate and its position, it can be reasonably assumed that Si doping reduce the strengthen of exciton transition resulting in a mixture of exciton transition and inter-band transition. This phenomenon that exciton transition is suppressed also happen in the case of undoped GaN thin films at high temperature^13,31^.

**Fig. 3:**
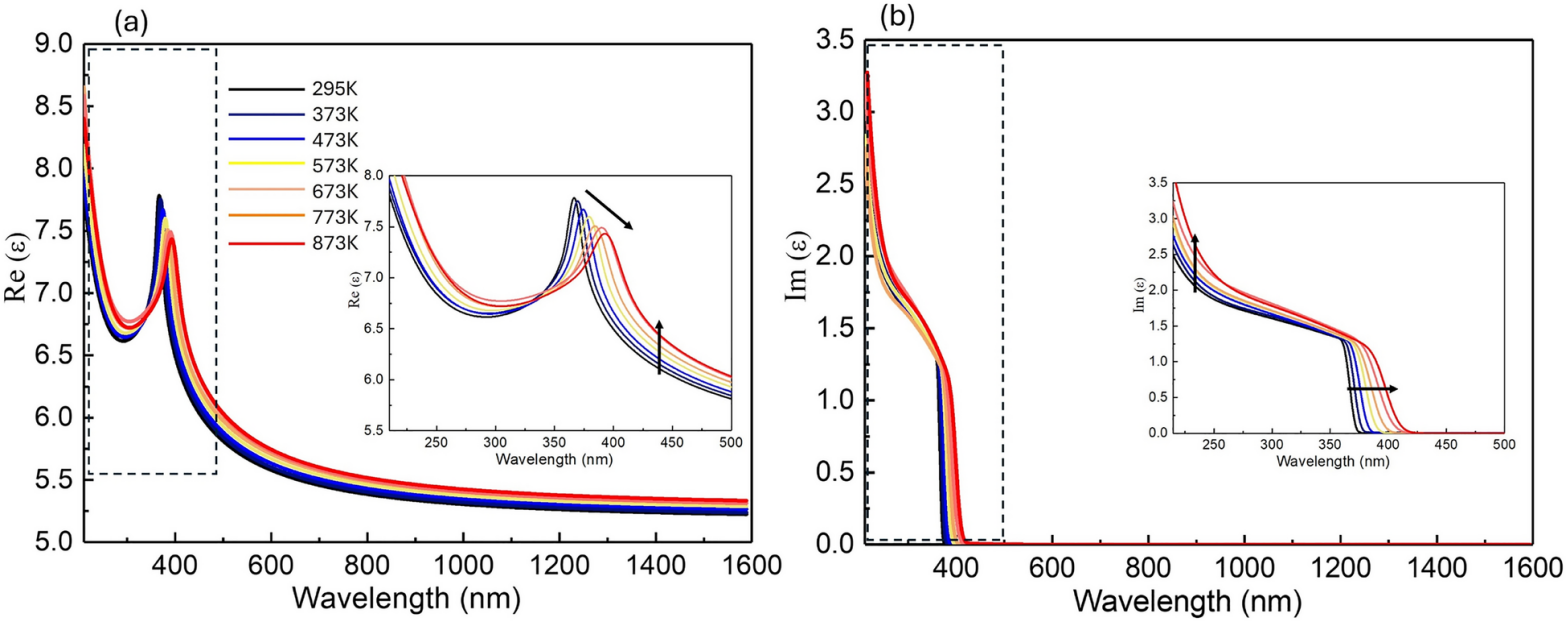
Real part (**a**) and imaginary part (**b**) of dielectric function $\varepsilon$ of silicon-doped GaN at elevated temperatures ranging from 298 K to 873 K. The inset provides a zoomed-in view of the spectral range highlighted by the black frame, with the black arrow indicating the trend of changes at elevated temperatures.

The temperature-dependent dielectric function $\varepsilon$ is presented in Fig. 3 for temperatures ranging from 298 K to 873 K. To highlight the variations in the dielectric function, the region within the dashed frame is magnified in the inset of Fig. 3. The changes in the dielectric function exhibit a consistent trend at elevated temperatures. The black arrow indicates the direction of these changes, showing a clear red-shift in the spectral position. This behavior can be explained by the increase in lattice vibrations (phonons) and thermal expansion as the temperature rises, leading to bandgap shrinkage. The reduction in the bandgap shifts the absorption edge, thereby affecting the refractive index. Additionally, as the temperature increases, the slope of the absorption edge becomes less steep, a phenomenon also attributed to strong electron-phonon interactions that broaden the exciton transition peak amplitude^13,31^.

To further clarify the movement of the transitions, the second derivatives $d^2\varepsilon /dE^2$ of these spectra is calculated and the standard analytic critical points (CPs) expression is employed to fit these data

2 $$\begin{aligned} \begin{aligned} \frac{d^2 \varepsilon }{d \omega ^2}&=n(n-1) A e^{i \phi }(E-E_0+i \Gamma )^{n-2} & n \ne 0, \\&=A e^{i \phi }(E-E_0+i \Gamma )^{-2} & n=0, \end{aligned} \end{aligned}$$

**Fig. 4:**
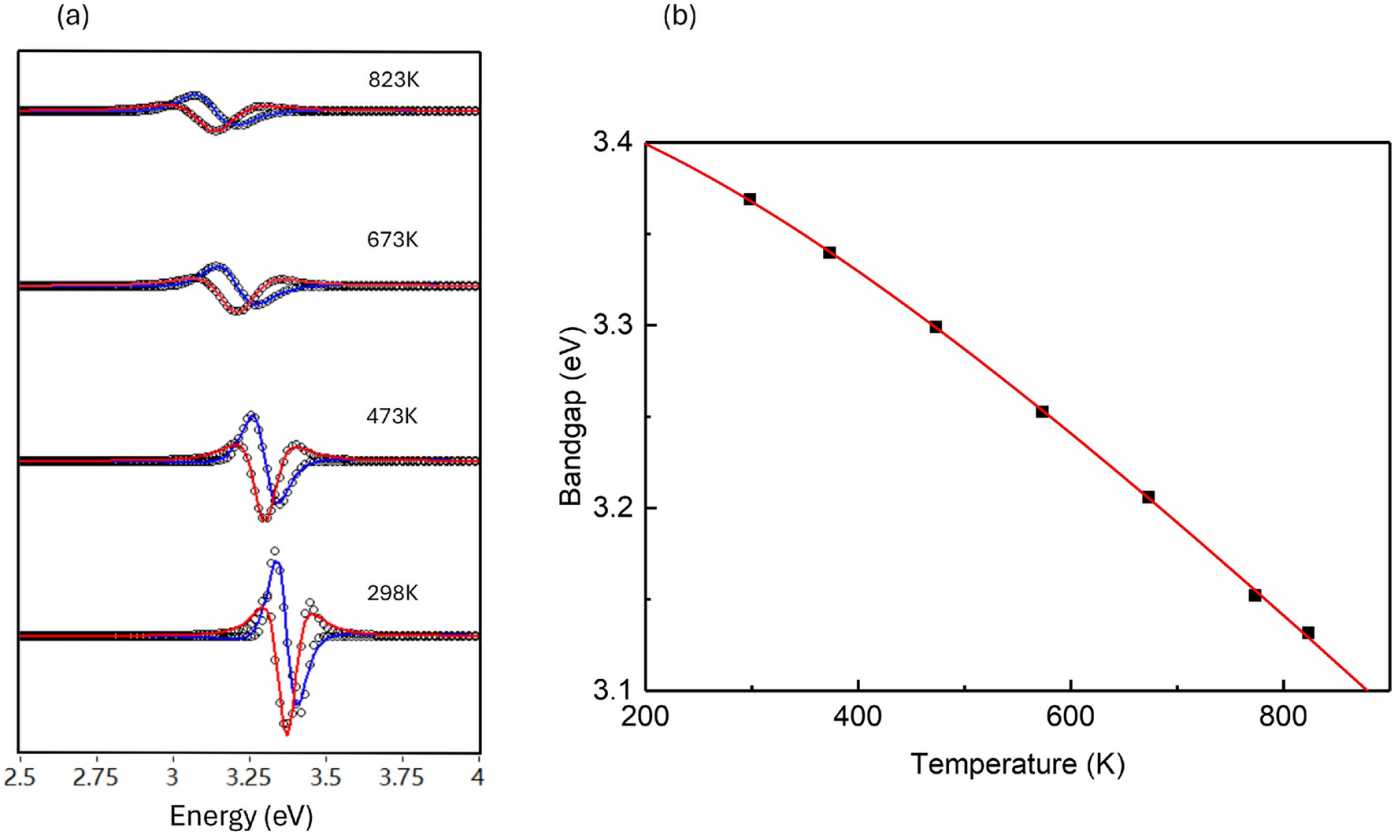
Exciton transition of Silicon doped GaN at elevated temperature from 298 K to 873 K. (**a**) the second derivatives $d^2\varepsilon /dE^2$ and fitted by Eq.(2). (**b**) Exciton transition fitted by Eq.(3).

In Eq. (2), the critical point (CP) is characterized by an amplitude $A$, a threshold energy $E_0$, a broadening parameter $\Gamma$, and a phase $\phi$. Both the real and imaginary parts of $\varepsilon$ were fitted simultaneously. Fig.4(a) shows the second derivatives of the experimental $\varepsilon$ data (open circles) and corresponding fits (lines) at elevated temperatures. The fundamental gap $E_0$ corresponds to a three-dimensional (3D) CP and the best fit is achieved with an exciton line-shape (n=-1), which is a common characteristic in CP analysis for many semiconductors^13,32^.

Fig.4(b) shows the temperature dependence of the $E_0$ derived from the second derivatives and it is further fitted with the empirical Varshni expression^33^. The empirical Varshni expression is frequently employed to optimize the performance of devices like laser diodes, LEDs, and solar cells as temperature-induced changes in the band gap significantly affect efficiency and output^34^.

3 $$\begin{aligned} E(T)=E_V-\frac{a T^2}{T+b} \end{aligned}$$

where $E_V$ representing the energy at 0 K is determined $3.431\pm 0.002$ eV. a is determined $6.337\times 10^{-4}\pm 0.051\times 10^{-4}$ eV/K reflecting the rate of energy with temperature. Considering in the initial attempt the fitted value of b was $567.79\pm 98.79$ K, the b value is fixed at the commonly used value of 600 K^35^, corresponding to the Debye temperature, which assumes this is the maximum phonon frequency in the material. All points locate at the fitting lines with coefficient of determination $R^\approx 1$. These value are smaller than that reported data of GaN thin films in the reference^13^ where $E_V=3.475\pm 0.002$ eV, $a=7.08\times 10^{-4}\pm 0.4\times 10^{-4}$ eV/K and the data from this work has smaller uncertainties.

In addition to the transition point, another critical parameter of semiconductor materials that attracts attention is the thermo-optic coefficient, which is influenced by factors such as crystal structure, expansions, and lattice defects. The thermo-optic coefficient of the GaN substrate is derived by calculating $\frac{dn}{dT}$ across different wavelengths, reflecting the variation of the refractive index with temperature. Refractive index n can be calculated using $n=\sqrt{\varepsilon }$ from Fig. 2. Considering the temperature-induced redshift in exciton and interband transitions, the refractive index changes near the bandgap in a complex manner^36^. Consequently, the thermo-optic coefficient is calculated for values from 400 nm to 1600 nm.

**Fig. 5:**
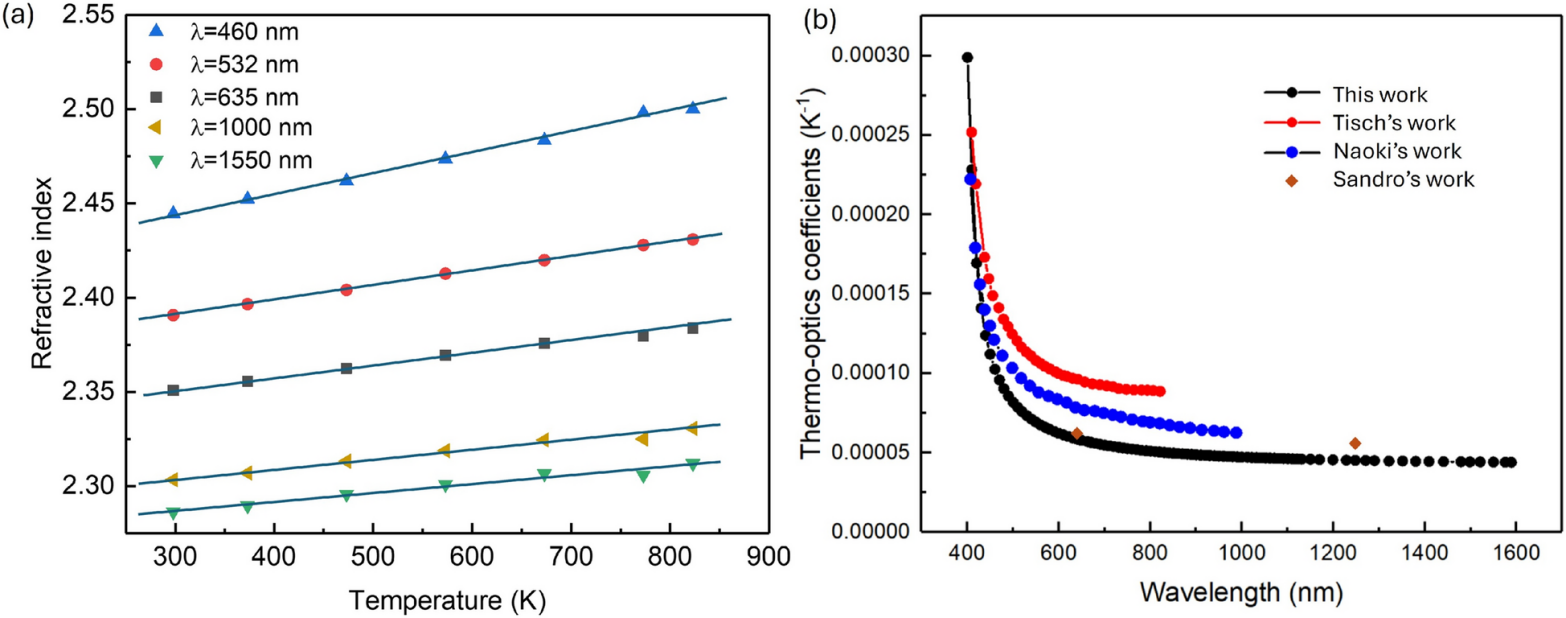
Evolution of refractive index at elevated temperature from 298 K to 873 K. (**a**) refractive index at different wavelengths. (**b**) Thermo optics coefficients fitted by Eq.(4) of this work and comparison with Tisch work^37^, Naoki work^38^, Sandro work^39^.

As shown in Fig. 5(a), five sets of refractive index $n$ values below the bandgap are selected for presentation. It is observed that $n$ varies almost linearly with temperature from room temperature to 873 K, implying that $n$ can be simply fitted by a first-order expression.

4 $$\begin{aligned} n(\lambda ,T)=\alpha (T-T_0)+n_0(\lambda ) \end{aligned}$$

Here, $\alpha$ with units of $\hbox {K}^{-1}$ represents the thermo-optic coefficient on the wavelength, $T_0$ is the reference temperature, and $n_0$ is the corresponding refractive index. The black spheres in Fig. 5(b) indicate the $\alpha$ fitted wavelength by wavelength over the range from 400 nm to 1600 nm. In the near-infrared region, $\alpha$ remains flat slopes at around 0.0005 but sharply increases near the bandgap. $\alpha$ is further parameterized using a Sellmeier model.

5 $$\begin{aligned} \alpha (\lambda )=A+\frac{B \lambda ^2}{\lambda ^2-C} \end{aligned}$$

$R^{2}$=0.999 indicates a good fit with the paramters of $A=1.396\times 10^{-5}\pm 0.004\times 10^{-5}$, $B=2.895\times 10^{-5}\pm 0.003\times 10^{-5}$ and $C=1.447\times 10^{5}\pm 0.002\times 10^{5} nm^{2}$. By virtue of Eq.(4), the refractive index can be inferred at any wavelength within the measurement range.

As comparisons, Fig. 5(b) also presents the thermo-optic coefficients from Tisch^37^ and Naoki^38^ measured from GaN thin films deposited on a substrate using ellipsometry. Across the entire wavelength range, their GaN values are larger than those determined in this study. At the wavelength of 632 nm, their thermo-optic coefficients is $9.683\times 10^{-5}$ $\mathrm K^{-1}$, $7.881\times 10^{-5}$ $\mathrm K^{-1}$ respectively and this work get $5.926\times 10^{-5}$ $\mathrm K^{-1}$. The discrepancy can be attributed to the facts that differences between thin film and single crystal substrate in doping concentration, material purity, and the higher defect density in thin film materials. Besides, The volume of the film expands during heating, affecting film thickness and surface state (such as surface roughness and oxide layer), which also impacts the accuracy of the thermo-optic coefficient. Sandro get a closer value to us $6.6\times 10^{-5}$ $\mathrm K^{-1}$ at the wavelength of 632 nm owing to the utilization of the commerical avaliable GaN substrate, but they took use of the principle of laser interference based on Fabry-Perot resonator resulting in a lack of the thermo-optics coefficients of the spectrum^39^. It is worth noting that the linear thermo optics coefficient only validated in the measured temperature range and it can not be simply expanded to the low temperature range because it was proved that nonlinear optical behavior of semiconductors is widely present in low temperature regions^31,40^.

## Conculsion

In summary, we report, for the first time, the dielectric function of Silicon doped GaN substrates from room temperature to 873 K over the wavelength from 250 nm to 1600 nm. Compared to thin film GaN materials, substrates exhibit a regular and consistent temperature dependence. Temperature dependent exciton transitions fit well with empirical Varshni expression. Additionally,the thermo-optic coefficient of GaN substrate is parameterize by a Sellmeier model over the wavelength range from 400 nm to 1600 nm. It is found that the temperature dependence of both the exciton transition and the refractive index of the substrate is lower than that reported in thin films, which can support the design and optimization of GaN-based optoelectronic devices.

## Data Availability

All data generated or analysed during this study are included in this published article and are available from the corresponding author on reasonable request.
